# Supplementary material for: Patterns of Intron Gain and Loss in Fungi
Source: PLoS Biol. 2004 Nov 30;2(12):e422. doi: 10.1371/journal.pbio.0020422 (PMC532390; doi:10.1371/journal.pbio.0020422)
Supplement: Table S1 — Also available at http://genes.mit.edu/NielsenEtAl/. (4.3 MB ZIP). [file pbio.0020422.st001.zip › NielsenEtAl/html/979.html]

AN0981.1.NCU08976.1.MG08904.1.FG06896.1


```
 CLUSTAL W (1.82) Multiple Sequence Alignments - Introns Inserted


Sequence 1: NCU08976.1	442 aa
Sequence 2: MG08904.1	475 aa
Sequence 3: FG06896.1	474 aa
Sequence 4: AN0981.1	534 aa
Alignment Length: 553 aa
Number Identitical Residues: 159 aa
Alignment Score (without introns) 7859


MG08904.1 	--------MASTGPIHWGLPSPALFSFPPKNVPDYIT-PISAQAHGSSAFVPPFHIPDNV
NCU08976.1	--------MAS---IFFSLPKASLFKFPPATNPGFVP-PPPAGPNSATVFN----IPDDV
FG06896.1 	--------MAEHFKLLGSMPDSSLFSFPPANAPAPIA-PLDVPTSIMRPFN----IPEQW
AN0981.1  	MYDLALDTGSGPAFVGFGLPPASLLKFPPDELPATVPAPLRDEPTWYQPFN----IPPGL
          	  . : .: :    :  .:* .:*:.***   *  :.:*    .     *     **   

MG08904.1 	YSAWLDAKVPITIAATYAITIKLLNAYNKSTNKRPWAISKTRAFFWFVVAHNVFLAVYSA
NCU08976.1	YYAWLDPKVPLTIATVYAVTAKLLNIYNKSTGKKPWAISKTRPFHWFVIAHNVFLAVYSA
FG06896.1 	FTAALDAKVPLTIAILYAVTVKSLNIYNKSNGKKPWAISKTRPFFAFVVLHNIFLCVYSA
AN0981.1  	YNQLLDIRVPITIASVYAVTVVILNRVNKSRGYKPYAFSQTRSFKIFVILHNVFLAVYSA
          	:   ** :**:***  **:*   **  *** . :*:*:*:**.*  **: **:**.****

MG08904.1 	WTWWGTLGMLRRSILSPTGPAGWAGTADSFCRVFGPPGVGNGIYYSDADHSWHSHAYGAS
NCU08976.1	WTFIGMLGAVRRSFVSPFGPEGLAGFVDSVCRINGPAGLSNSVFYNDEAGAFQTYSPHVT
FG06896.1 	WTFWGMLSGMRRSIQNPTGPQGLAGTADSFCRLHGASGLGNSVYYNETSNSFVSATEHAA
AN0981.1  	WTFAGMVRAFRYTLP-AWQENGLVGIVDALCKCNGPRGYGNAATYDTTINKWSIQNPEYR
          	**: * :  .* ::  .    * .* .*:.*:  *. * .*.  *.     :        

MG08904.1 	LSAG--PDRTTSGRMWNEGLAFYGWLFYISKFYEVVDTLIIIAKGKLSSTLQTYHHAGAM
NCU08976.1	LGADGNPSRFDSGRLWNEGLAFYGWIFYLSKFYEVVDTLIILAKGKLSSTLQTYHHAGAM
FG06896.1 	IING-LPSGTVGGRMWNEGLAFYGWIFYLSKFYEVLDTFIILAKGKLSSTLQTYHHAGAM
AN0981.1  	LAEGGVPDSTDVGRLWNQGLAFLGWIFYLSKFYEVLDTAIILAKGKKSSTLQTYHHAGAM
          	:  .. *.    **:**:**** **:**:******:** **:**** *************

MG08904.1 	MCMWAGMRYMAVPIWTFLFFNSFIHAMM0YTYYTLTAFHIKVPVFIKRSLTTMQITQFVV
NCU08976.1	LCMWAGMRYMSTPIWVFCFVNSFIHALM0YTYYTVTAFNIRVPTPIKRTLTSMQITQFLL
FG06896.1 	LCMWAGMRYMSAPIWQFVLINSFIHSLM0YFYYTLTAFSIRVPTPIKRSLTTMQITQFIV
AN0981.1  	MCMWAGIRYVAPPIWIFTLVNSAIHAMM0YTYYTLTALRVRVPTIVKRSLTTMQITQFVI
          	:*****:**:: *** * :.** **::* * ***:**: ::**. :**:**:******::

MG08904.1 	GASYAMVHSFVSYTIPVALNGNPVTAAPGAAATPAGAATTNQA-IIDNMKNMIGGLVGSK
NCU08976.1	GASYAIVHSFVSYTIPVVVAN------PEKTASAVEAAGT----VVDSQKVLVFG-----
FG06896.1 	GASNTIAYSFISYKVPVAIIQKLSVPAYATTAAPDASQETVADGAVESIKKFIWG--AAE
AN0981.1  	GSAMAASYLFVYYTLPPVRFV------PTTSAAPVVAATG-----LSWVKQVAFR---AA
          	*:: :  : *: *.:* .            :*:.  :        :.  * .      : 

MG08904.1 	PATAADAAPSGMYRVAGLSDTTTYGQRVVPCISSTGETFAIWLNVLYLAPLTYLFVMFFI
NCU08976.1	NATRVDAADA------------TYGQRVVPCVTSNNSTFAIWLNVFYLAPLTYLFISFFI
FG06896.1 	GAAAPGAAPVASQAVTSEAFTAETQYVTQPCIMTTGETFAIWLNVLYLAPLTYLFVSFFI
AN0981.1  	GAEGIAENVGHVENNLGAPREVVQPGPMVTCMDTSGQGFAIYLNVAYLLPLTYLFARFFV
          	 *              . .          .*: :... ***:*** ** ******  **:

MG08904.1 	SSYLRRSS-------VDAARFK--------------------KGTSARRESNVALAEKAR
NCU08976.1	ESYTRRS---------NAAEKT--------------------KNTKP--VDNVAMAEKAG
FG06896.1 	ASYVKRS---------NAASKK--------------------SHDVN---ANIALAEKAG
AN0981.1  	RSYLYRKEPGPQATHIHAAEKAGLDALKGVSREIQKAAEMSGETSETTEDEAIAKSRSTR
          	 **  *......::  .**   . .: .. : . ..::. :..         :* :..: 

MG08904.1 	LGGCPSSREGGLWWRG----CSGSHEWEWKGEE----------IKWCRR-----------
NCU08976.1	WEAAKK-MEGEVYGET----KEDTYEARTTRSA----------SKRTLRQRA--------
FG06896.1 	WDAAKG-IEKEIYDGANTANGSSADEASRASTP----------KKANGKSRRKA------
AN0981.1  	SHVQDTVDNSPIRTRSSAAKKARATESQREPEQGFSTVPAGKGAKRISKEETQSAGGPAE
          	        :  :    .::.   : *       . :: .:... *   :.  .::...:.

MG08904.1 	--------------
NCU08976.1	--------------
FG06896.1 	--------------
AN0981.1  	VKTSNPFGVLGNSN
          	 .::.. .  ..:.
```
